# An Algorithmic Approach to the Management of Infantile Digital Fibromatosis: Review of Literature and a Case Report

**Published:** 2018-05-07

**Authors:** Elizabeth H. Eypper, Johnson C. Lee, Ashley J. Tarasen, Maxene H. Weinberg, Oluwaseun A. Adetayo

**Affiliations:** ^a^Albany Medical College, Albany, NY; ^b^Division of Plastic Surgery; ^c^Department of Pathology, Albany Medical Center, Albany, NY

**Keywords:** infantile digital fibromatosis, fibromatosis, pediatric extremity mass, foot mass, extremity tumor

## Abstract

**Objective:** Infantile digital fibromatosis is a rare benign childhood tumor, infrequently cited in the literature. Hallmarks include nodular growths exclusive to fingers and toes and the presence of eosinophilic cytoplasmic inclusions on histology. This article aims to exemplify diagnoses of infantile digital fibromatosis and possible treatment options. **Methods:** A computerized English literature search was performed in the PubMed/MEDLINE database using MeSH headings “infantile,” “juvenile,” “digital,” and “fibromatosis.” Twenty electronic publications were selected and their clinical and histological data recorded and used to compile a treatment algorithm. **Results:** A 9-month-old male child was referred for a persistent, symptomatic nodule on the third left toe. A direct excision with Brunner-type incisions was performed under general anesthesia. The procedure was successful without complications. The patient has no recurrence at 2 years postsurgery and continues to be followed. Histological examination revealed a proliferation of bland, uniformly plump spindle cells with elongated nuclei and small central nucleoli without paranuclear inclusions consistent with fibromatosis. **Conclusions:** Asymptomatic nodules should be observed for spontaneous regression or treated with nonsurgical techniques such as chemotherapeutic or steroid injection. Surgical removal should be reserved for cases with structural or functional compromise.

Infantile digital fibromatosis (IDF) is a rare, benign tumor of myofibroblasts occurring on the fingers and toes in early childhood. As a rare entity, available literature describing diagnostic criteria and treatment options for IDF is proportionally scarce. Reye^1^ first classified these tumors in 1965, noting that they can be distinguished from other fibrous growths on the basis of their common anatomic location, presenting age group, and pathology. Infantile digital fibromatosis typically appears as singular or multiple nodules on the digits within the first year of life but may also be present at birth in up to one third of patients.^1^^-^3 The nodules are typically firm, less than 2 cm in diameter, and often painless, although some cases present with decrease in or loss of joint function or discomfort.^2^^-^9 Larger tumors may additionally begin to compromise adjacent digits with long-term functional consequences.^7^^,^^10^

Histopathological diagnosis is usually based on a few characteristic findings: cellular proliferation restricted to the dermis; interlacing fascicles extending to the epidermis; and tumor cells surrounding the adnexal structures.^3^ The most pathognomonic feature is the presence of eosinophilic paranuclear inclusions consisting of an actin aggregate, although they are not necessary for a diagnosis of IDF if the clinical picture and other histological findings are standard.^3^

Treatment of IDF has also been varied, owing to a paucity of data on remission rates and different treatment modalities. Numerous case reports have demonstrated success with either surgical excision of nodules or a more conservative observational approach. Surgical intervention has been widely effective, although recurrence remains a problem in up to 61% to 74% of cases.^2^^,^^3^ Additional studies reporting on more aggressive excision techniques have nevertheless shown a lower recurrence rate, closer to 13% to 36%.^11^^-^14 Other approaches to treatment have included topical steroids, intralesional steroids, and intralesional 5-fluorouracil.^6^^,^^15^^,^^16^

Here, we describe a case of IDF in a 9-month-old male child presenting with a painful nodule on the dorsum of his left third toe, summarize case management, and review the available literature to provide a management algorithm for IDF (Table 1).

## METHODS/CASE REPORT

A 9-month-old otherwise healthy male child was referred for evaluation of a spontaneously occurring nodule on the dorsal aspect of the third toe proximal and middle phalanges, noticed 2 months earlier. On examination, the nodule was hard and ill-circumscribed, with significant blanching and thinning of the overlying skin. The nodules were reported to be tender, and the family noted increasing discomfort with shoes. A similar lesion had developed 2 weeks prior to evaluation over the middle phalanx of the same toe. The patient's hands and the rest of the feet were otherwise normal. The patient's medical history was negative for trauma, infection, or signs of excoriation. Family history was otherwise unremarkable, except for a history of Irish and Scottish descent on both parental histories. Radiographs of the lesions demonstrated no bony erosions, osteophytes, or any other radiographic evidence of bony pathology or involvement. Because of the overlying skin compromise secondary to the compressive effect of the nodules, and the discomfort and irritation with shoe fit and friction, the patient's family elected to undergo operative intervention at 11 months of age.

## RESULT

**Figure 1:**
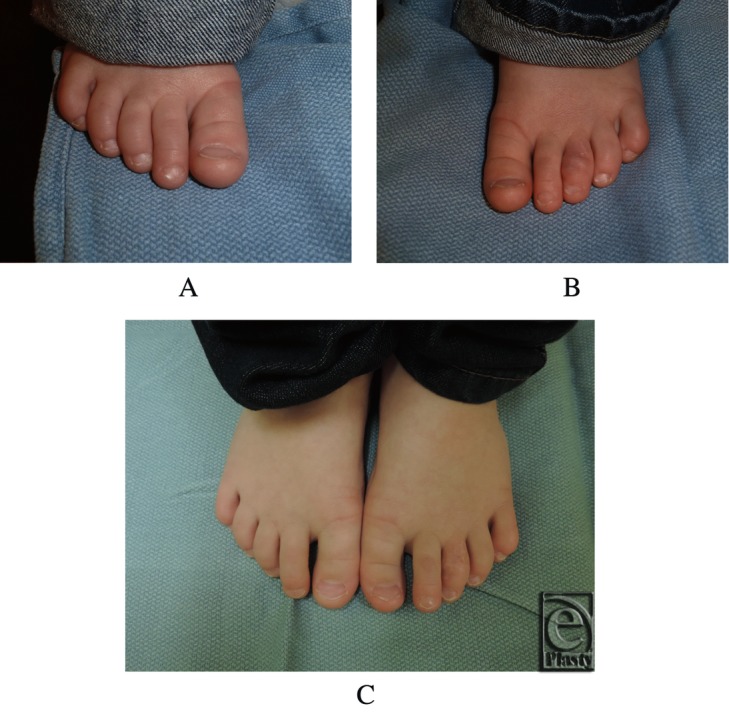
Postoperative photographs taken at 1 and 2 years follow-up. The (a) right and (b) left feet at 1 year follow-up. (c) Both feet at 2 years follow-up.

**Figure 2:**
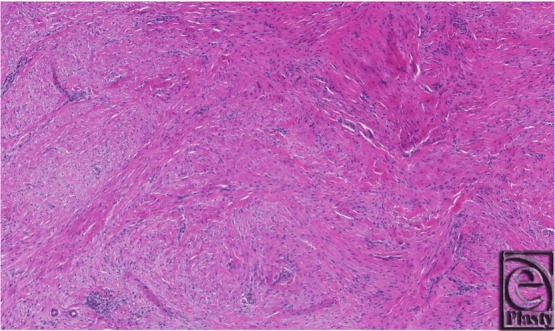
Hematoxylin-eosin stain of tissue removed from the foot of a 9-month-old patient with infantile digital fibromatosis. Spindle cells demonstrate elongated nuclei with small central nucleoli. Paranuclear inclusions are not present.

A direct excision of the lesions with Brunner-type incisions was performed under general anesthesia. The lesion was noted to abut and was adherent to the underlying dermis from which the extirpation was performed. The procedure was successful without complications. The patient is now 33 months of age, has no recurrence at 2 years postsurgery, and continues to be followed (Fig 1). Histological examination revealed a proliferation of bland, uniformly plump spindle cells with elongated nuclei and small central nucleoli without paranuclear inclusions (Fig 2). Variably admixed collagen was identified. Immunohistochemical studies showed the tumor cells to be positive for smooth muscle myosin, desmin, CD10, and calponin, consistent with fibromatosis.

## DISCUSSION

Reye^1^ first proposed IDF as a rare but distinct group of childhood fibromatoses in 1965. Between 1965 and 1991, there were approximately 200 cases of IDF described in the literature. Although additional cases have been described since, IDF remains a rare disease, comprising only about 2.5% of all fibromatoses.^17^ Nodules may be present at birth or may develop in early childhood, with up to 86% occurring within the first year of life.^2^ Single lesions typically occur on the dorsal or lateral aspects of the digits, sparing the thumb and great toe.^1^^-^3^,^^10^ However, some cases present with multiple nodules on the same or different digits.^1^^,^^3^^,^^6^^,^^8^^,^^11^^,^^14^^,^^15^^,^^18^^-^24 The lesions are usually smooth and dome-shaped, with flesh-colored to red overlying skin. A minority may also present as polypoid or ulcerated lesions.^3^ These tumors initially exhibit a period of slow growth when they first appear, followed by a period of rapid growth extending 10 to 14 months before the lesion stabilizes and regresses.^25^^,^^26^ Numerous reports have documented that spontaneous involution may occur over a period of months to years following the stabilization phase, with most noting total regression in 1 year (range, 6 months-5 years).^5^^,^^7^^,^^8^^,^^18^^,^^19^^,^^21^^,^^27^ No metastasis has ever been reported.

The differential for IDF includes juvenile aponeurotic fibroma, pachydermodactyly, keloids, terminal osseous dysplasia, and pigmentary defects.^8^ A positive family history should be considered closely, in addition to any history of trauma in the patient. Juvenile aponeurotic fibroma, also known as calcifying fibroma or juvenile palmoplantar fibromatosis, is an invasive tumor characterized by calcification. Infantile digital fibromatosis is noninvasive, marking a clear difference between the two. Lesions in juvenile aponeurotic fibroma are most common on the palm/sole, as opposed to IDF, which is limited to the digits. This disease also differs from IDF in that the overlying skin is nonadherent, whereas skin is generally adherent in infantile digital fibromas. Pachydermodactyly may be distinguished from IDF by the patient population affected—typically young adult males. These lesions may persist, unlike IDF that shows a tendency to spontaneously involute. In addition, pachydermodactyly frequently results from rubbing or mechanical injury, which may be notable from the patient's history. Terminal osseous dysplasia and pigmentary defects have been identified as affecting only females, due to an autosomal dominant inheritance pattern. Lesions may occur on the digits but are also common on the face and scalp. These lesions may appear punched-out, as opposed to the dome-like nodular appearance of IDF. Distal limb and eye abnormalities may also be seen. Differentiation of a number of other rare juvenile fibromatoses can usually be established on the basis of clinical characteristics, histology, and immunohistochemical markers.^10^

In a histopathological review of 12 patients with 18 tumors, Reye described IDF nodules as frequently less than 1 cm in diameter, causing flattening of rete ridges, acanthosis, and compression atrophy despite an intact epidermis. Interlacing bundles of fibrocellular material were found within the dermis. Individual cells demonstrate few mitotic figures, and lightly eosinophilic inclusion bodies can be located near one pole of the nucleus with hematoxylin-eosin staining. Treatment with phosphotungstic acid–hematoxylin stains these characteristic inclusions deep purple, allowing more ready identification. Reye examined 40 sections from pediatric fibrosarcomas and other benign fibromatoses with identical staining techniques without identifying paranuclear inclusions, confirming IDF as an independent entity.

These characteristics have since been reiterated and elaborated upon by Laskin et al.^3^ Three findings considered necessary for a diagnosis of IDF include cellular proliferation that is restricted to the dermis, causing dome-shaped or polypoid nodules; perpendicular fascicles extending directly beneath the skin, consisting of spindle-shaped myofibroblasts and collagen; and the presence of tumor cells that surround adnexal structures and infiltrate the periadnexal adipose tissue.^3^ Masson's trichrome stain is the criterion standard for identifying the paranuclear inclusions that are pathognomonic for IDF. When present, inclusion bodies appear bright red with trichrome stain and are identifiable in up to 80% of tumors.^3^ A recent report indicates that fine-needle aspiration cytology is sufficient to uncover inclusion bodies, providing a less invasive biopsy method for young IDF patients.^28^ Although pathognomonic, these inclusion bodies as first described by Reye are not necessary for a diagnosis of IDF if the clinical picture and other histological findings are standard.^3^ As in our case, Laskin et al^3^ and others^20^^,^^29^ have described specific staining patterns for α-smooth muscle actin, desmin, and calponin as confirmatory measures in the diagnosis of IDF, which are particularly helpful when cytoplasmic inclusions are absent. It is also possible that the absence of inclusion bodies indicates fibrous scar formation and tumor regression.^24^^,^^30^

Given the rarity of IDF, there is an inherent challenge in diagnosis and attempting to define a treatment protocol. Surgery was previously considered the primary therapy for IDF but is now advocated as a therapy of last resort for cases that present with symptoms such as pain or joint deformity.* The recurrence rate following surgery is high and has been estimated at 61% to 74%.^2^^,^^3^ The median time to recurrence in one study was 4 months and may be attributable to incomplete surgical margins, as reported by others who advocate wide surgical excision or Mohs micrographic surgery.^2^^-^4^,^^10^^-^14^,^^22^^,^^34^^,^^35^ Additional studies have noted that lesions may recur in multiple, or even larger and more aggressive, forms than the initial nodule, as surgical trauma to the fibroblasts may initiate new growth.^2^^,^^6^^,^^9^

Nonsurgical approaches to treatment of disabling lesions have included intralesional triamcinolone injections of 0.5 to 2 mL at 10 mg/mL with 1 to 3 injections over the course of 5 to 40 months, which showed promise in a pilot study.^6^ Topical imiquimod, in comparison, may cause irritation with no clear improvement in lesion size.^9^^,^^15^ Another study used intralesional injections of 0.2 mL 5-fluorouracil at 50 mg/dL injected at 2 to 3 sites, monthly for 5 months, to treat a patient with no reported adverse systemic effects.^16^ However, injections may cause significant pain, particularly when repeated injections are required.^6^^,^^16^ For cases in which there is no pain or functional deficit, a watch-and-wait approach is advocated.^†^

## MANAGEMENT ALGORITHM

**Figure 3:**
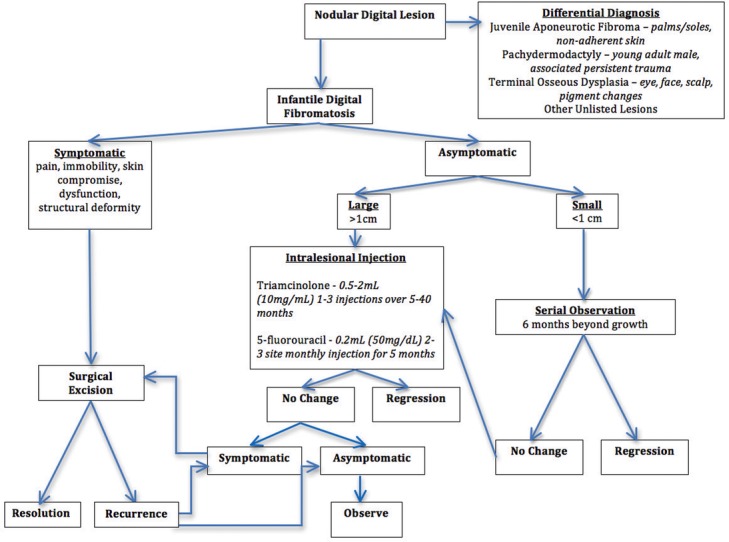
Algorithm for the management of patients diagnosed with infantile digital fibromatosis.

The authors propose a management algorithm for patients diagnosed with IDF. When a nodular lesion clinically consistent with IDF is discovered in a pediatric patient, the risks and benefits of operative management versus observation should be considered. None of the injection treatment methods have been explored in large studies, to date, leaving surgery as the most accepted treatment of symptomatic lesions. However, in the subset of asymptomatic patients with relatively large-sized tumors (>2×2 cm), preemptive treatment with intralesional triamcinolone or 5-fluorouracil should be considered when tolerated.^16^ Given reports of spontaneous regression, asymptomatic IDF lesions can be conservatively managed with routine follow-ups and will therefore be the recommended approach to treatment in most cases. The proposed treatment algorithm is shown in Figure 3.

Infantile digital fibromatosis is a rare childhood disease of benign myofibroblast tumors occurring on both upper and lower digits. Nodules that are asymptomatic should be watched over time for spontaneous regression or may be treated with chemotherapeutic agents, steroids if they become large or unwieldy. Surgical treatments should be reserved for cases with structural or functional compromise to minimize trauma and the associated risk of recurrence.

## Figures and Tables

**Table 1:** Overview of case presentations and outcomes from cited literature*

Author (Year)	No. of nodules	Inclusion bodies	Resolution with excisions	Resolution with observation	Resolution with other treatment^†^	Author recommendation
Albertini et al^4^ (2002)	1	+	+			Conservative or Mohs micrographic surgery if necessary
Azam and Nicholas^18^ (1995)	3	+	+	+		Conservative treatment
Beckett and Jacobs^2^ (1977)	3	+	+	NS		Conservative or wide margin if necessary
Braun and Helbig^19^ (2014)	2	+	+	+		Conservative treatment w/follow-up
Burgert and Jones^23^ (1996)	7	NS		+		Conservative treatment
Campbell and Petrick^35^ (2007)	1	+	+			Mohs micrographic surgery for large tumors
Chirayil et al^31^ (2001)	1	+	+			Surgery only for impairment
Failla et al^15^ (2009)	3	+				Surgery only for impairment
Falco and Upton^11^ (1995)	15	+	+			*Wide excision*
Girgenti et al^12^ (2012)	5	+	+	+		Surgery only for impairment
Hayashi et al^24^ (1995)	2	+	+			Limited surgery for impairment
Holmes et al^6^ (2011)	12	NS	+		+	*Intralesional steroids*
Kang et al^30^ (2002)	1	+		NS		Surgery only for impairment
Kanwar et al^32^ (2002)	1	+		NS		Surgery only for impairment
Kawaguchi et al^27^ (1998)	1	+		+		Surgery only for impairment
Khan et al^33^ (2001)	1	+	+			Limited surgery only for aggressive tumors
Laskin et al^3^ (2009)	74	+^‡^	NS	NS		Surgery only for impairment
Liu et al^34^ (2014)	1	+	+			*Complete resection*
Netscher et al^7^ (2001)	1	NS	+	+		Joint/tendon-sparing surgery if necessary
Netscher et al^10^ (2009)	2	+	+			Wide excision if necessary
Niamba et al^8^ (2007)	5	NS	+	+		Surgery only for impairment
Oh et al^16^ (2005)	1	+			+	*5-fluorouracil warrants further investigation*
Paloni et al^21^ (2013)	2	+		+		Avoid surgical intervention
Spingardi et al^22^ (2011)	15	NS	+			*Wide excision*
Talbot et al^14^ (2007)	7	+	+	+		*Complete resection*
Taylor et al^9^ (2008)	1	+	+			Avoid aggressive treatment

*The number of nodules is recorded to account for multiple nodules in some patients. Positive treatment outcomes are recorded as present or absent, owing to the fact that some nodules required multiple rounds of the same, or different, treatments in order to attain complete resolution. Article-specific management recommendations are noted. The minority, advocating either all-encompassing surgical resection or pharmacological treatment, are indicated by italic fonts. NS indicates not specified.

^†^Other modalities include intralesional triamcinolone and 5-fluorouracil, specified under Recommendation.

^‡^57/74 nodules.

## Footnotes

* *References 2-5, 7-10, 14, 15, 18, 19, 21, 24, 27, 30-33*.

† *References 2-5, 7-10, 14, 15, 18, 19, 21, 23, 27, 30-32*.
